# Rice husk valorisation by *in situ* grown MoS_2_ nanoflowers: a dual-action catalyst for pollutant dye remediation and microbial decontamination†

**DOI:** 10.1039/d4ra00862f

**Published:** 2024-04-16

**Authors:** Rahul Ranjan, Smruti B. Bhatt, Rohit Rai, Sanju Kumari Sharma, Rishabh Ranjan, Ankit Bharti, Prodyut Dhar

**Affiliations:** a School of Biochemical Engineering, Indian Institute of Technology (BHU) Varanasi Uttar Pradesh-221005 India prodyut.bce@iitbhu.ac.in; b Department of Biotechnology, National Institute of Technology Durgapur West Bengal 713209 India

## Abstract

Rice husk (RH) is a common agricultural waste generated during the rice milling process; however, a major portion is either burned or disposed of in landfills, posing significant environmental risks. In this study, RH waste was transformed into bio-based catalysts *via* delignification cum *in situ* growth of MoS_2_ (DRH-MoS_2_) for efficient pollutant dye removal and microbial decontamination. The developed DRH-MoS_2_ exhibits nanoflower-like structures with a 2H-MoS_2_ phase and a narrow band gap of 1.37 eV, which showed strong evidence of photocatalytic activity. With the presence of abundant hydroxyl functionality, delignified rice husk (DRH) exhibits a malachite green (MG) dye adsorption capacity of 88 mg g^−1^. However, *in situ* growth of MoS_2_ nanosheets on DRH enhances MG degradation to 181 mg g^−1^ under dark conditions and 550 mg g^−1^ in the presence of light. Mechanistic insights reveal a synergistic adsorption-*cum*-degradation phenomenon, amplified by generation of reactive oxygen species during photodegradation which was confirmed from radical scavenging activity. Interestingly, DRH-MoS_2_ demonstrates potent antibacterial activity against *Staphylococcus aureus* (*S. aureus*) and *Escherichia coli* (*E. coli*) with sustained photodegradation efficiency (>80%) over three cycles. The present work reports a cost-effective and scalable strategy for environmental remediation of real wastewater which usually contains both dye pollutants as well as microbes using abundantly available renewable resources such as sunlight and agricultural biomass wastes.

## Abstract 2

In this study, we carried out valorization of rice husk through *in situ* growth of MoS_2_ nanoflowers for simultaneous pollutant dye remediation and microbial decontamination.

## Graphical abstract

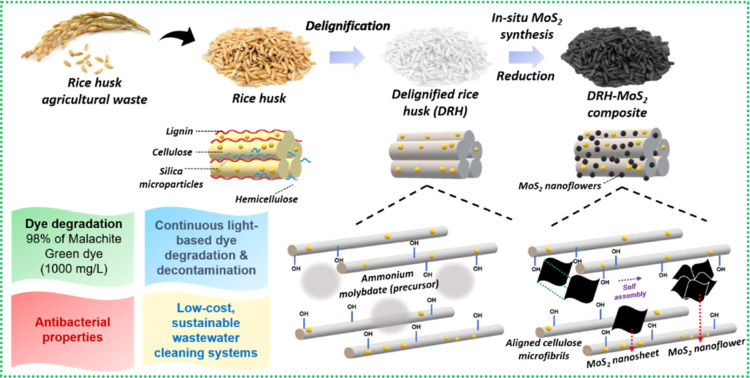

## 1. Introduction

As one of the most produced and consumed crops, rice feeds more than 40% of the world's population; however, it results in the generation of large amounts of waste globally (∼731 million tons of rice straw and ∼160 million tons of RH).^1–4^ India is a major agriculture-based economy, with rice as one of the major crops with an annual yield of 124 million metric tons, generating RH as waste, accounting for ∼20% of the crop portion.^5^ RH is an abundantly available by-product from the rice milling industry, with an annual production of 120 million tons with limited applications.^6^ It has been used as animal feed but has challenges due to its poor nutritional value,^7^ high fibre content,^8^ and allergic effects in the digestive tract.^9^ RH has a tough, water-insoluble matrix composed of cellulose–silica structures with the composition of cellulose (35%), hemicellulose (25%), lignin (20%), and ash with a silica content of 15–20%.^10^ RH is highly porous and lightweight due to high ash content with a bulk density of 96–160 kg m^−3^ and 75–90% organic matter. The exterior part of RH is made of dentate rectangular elements of silica with thick cuticles and surface hairs.^11^ RH generated from the processing of rice grains is usually disposed of by landfill, drastically increasing the aesthetic pollution, eutrophication, and perturbations in the aquatic ecosystems and organisms. Due to its high volume, RH is difficult to handle through proper waste management. It is generally subjected to uncontrolled burning, which generates ashes, contributing to air pollution and global greenhouse gas emissions. Due to high accumulation and potential ecological toxicity, strategies for using RH biomass for innovative applications are the current need of the hour.

In recent years, researchers have chemically modified RH waste for a plethora of applications such as pest control,^12^ fertilisers,^13^ insulators,^14^ construction,^15^ catalysts,^16^ and as adsorbents.^17,18^ Due to its low cost, abundance, high porosity, functionality, and surface area, RH makes it a suitable substrate as a high-performance adsorbent for removing carcinogenic pollutants, dye, and heavy metals. RH has been used as adsorbents in three forms, firstly (i) as direct RH,^19–21^ secondly (ii) as production of biochar through thermal processing^22,23^ and thirdly (iii) through strategic chemical modifications.^24–26^ Based on the first approach, Amin *et al.*^27^ utilised unmodified RH to remove arsenic (As) from groundwater and achieved 96% As removal at 595 μg L^−1^ concentration. Kumar *et al.*^21^ also utilised RH to remove cadmium (Cd) with an efficiency of 98.65% at 25 mg per L Cd concentration. Based on the second approach, thermal processing was used to produce RH biochar. Leng *et al.*^28^ produced high surface area biochar (21.7 m^2^ g^−1^) using water/ethanol mixtures processed hydrothermally at 260–340 °C, which shows maximum MG dye adsorption capacity of 67.6 mg g^−1^ following pseudo-second-order kinetics. Tsai *et al.*^29^ prepared mesoporous biochar from rice husk to remove carcinogenic MG (at 30 mg L^−1^) and achieved an efficiency of ∼96.96%.^30^ also utilised rice husk biochar for the removal of two cationic dyes, Basic blue 41 and Basic Red 06 (at 50 mg L^−1^), with adsorption efficiency of ∼80% and based on the third approach of RH chemical modification,^31^ modified RH with cationic hydroxypropyl octadecyl dimethylammonium, which aided in the removal of Congo Red, Acid Black 24, Diamine Green B with adsorption capacity of 580.09, 268.88, and 207.15 mg g^−1^ respectively. Mechanistic investigation shows strong hydrogen bonding and electrostatic interactions between sulphite groups in dye and ammonium groups of cationic RH, resulting in improved dye remediation. Chakraborty *et al.*^25^ performed alkali (NaOH) modifications to RH and employed it to remove crystal violet dye with 98.17% efficiency for 50 mg L^−1^ dye concentration. However, due to the presence of silica and lignin in its backbone along with cellulose, RH forms rigid structures, making it challenging to modify chemically, so higher dye remediation efficiency is not achieved. A potential approach to remediate dye is the introduction of active catalysts with dye-degrading capabilities, which can improve remediation efficiency and adsorbent reusability. To overcome the limitations of RH, we chemically delignify RH followed by *in situ* growth of catalytically active molybdenum disulfide (MoS_2_), which is expected to improve dye degradation efficiency. MoS_2_, due to its attractive properties such as visible light adsorption and photocatalytic action *via* electron–hole pair recombination, has been abundantly utilised for the photodegradation of dyes and wastewater pollutants.^32^ MoS_2_ is a 2D, covalently linked with a band gap of 1.96 eV, making it suitable for visible light absorption and catalysis. The polluted industrial wastewater is generally composed of physical, chemical, and biological contaminants with mixtures of dust particles, dyes, microbes, *etc.*^33^ Most studies reported in the literature only focus on the remediation of dyes or pollutants. However, many pathogenic microbial loads remain unprocessed and require additional pre-treatment steps to make water suitable for consumption and reuse. MoS_2_ generates active free radicals, which mediates the degradation of contaminants and simultaneously forms ROS, which induces antimicrobial properties.^34^ For examples in the recent study, Qiu *et al.* had grown MoS_2_ into the highly porous structure of cellulose *via* hydrothermal process for removal of Congo Red (334.92 mg g^−1^).^35^ The *in situ* formation of 2H-MoS_2_ nanoparticles occurred on bacterial cellulose, leading to the degradation of MG (100% for 10 mM) and bacterial disinfection against *E. coli* and *S. aureus*.^36^ In a study by Gao *et al.* magnetic cellulose microparticles served as carriers for the *in situ* growth of MoS_2_ which demonstrated maximum adsorption capacity of 469.5 mg g^−1^ specifically for Hg^2+^.^37^ In a study by Zhao *et al.*, growth of MoS_2_ nanoparticles within the nanofibrous structures of porous cellulose led to the adsorption of 327.6 mg g^−1^ of Pb^2+^ and removal of 80% of Congo Red and methylene blue.^38^ Recent studies addressing the adsorption and photodegradation of dyes through the use of MoS_2_ and its composites were summarized in Table S8.†

The current work presents the fabrication of *in situ* grown delignified DRH-MoS_2_ with high catalytic activity for photodegradation of MG dye along with decontamination of microbial load. RH was delignified to remove the aromatic lignin present, exposing the hydroxyl groups of cellulose–silica structure for strategic fabrication and growth of MoS_2_ with high loading fractions. Interestingly, the growth of MoS_2_ nanosheets through hydrothermal treatment in the confined cellulose interlayers of RH resulted in self-assembly to form MoS_2_ nanoflowers. Mechanistic studies were carried out to understand the combinatorial approach of dye adsorption and degradation by DRH-MoS_2_, which synergistically improved dye removal efficiency. The current approach for valorising rice waste into high-performance catalytically active adsorbents will overcome the issues related to its disposal. Furthermore, DRH-MoS_2_, with continuous dye remediation capabilities, microbial decontamination, and high recyclability, shows promising applications in developing industrially scalable chemo-biological wastewater remediation technologies.

## 2. Materials and methods

RH was purchased from Navya Agriculture and Allied Products, India. Ammonium molybdate tetrahydrate (99% purity), MG, and sodium chlorite (98% purity) were procured from Hi-Media Laboratory, India. Glacial acetic acid (99% purity), thiourea (98% purity), and sodium acetate trihydrate (98% purity) were purchased from Sisco Research Laboratories (India). All the chemicals were utilised without further purification, and the experiments were performed with Millipore water (Merck, India, 0.66 MΩ cm conductivity).

### 2.1 Delignification of RH

Firstly, acetate buffer (pH – 4.8) was prepared by mixing acetic acid (2.4 v/v%) and sodium acetate trihydrate (5.4 wt/v%) in distilled water, followed by dissolving 3 wt% of sodium chlorite flakes to prepare the dignifying solution. RH was mixed in the prepared solution and incubated at 85 °C for 18 hours. After the incubation, the solutions were discarded, and the delignified husk was washed five times with distilled water to remove unreacted chemicals or impurities, followed by drying at 60 °C in a hot air oven.

### 2.2 *In situ* growth of MoS_2_ on DRH

The delignified RH was soaked for 2 hours in a 40 mL solution of ammonium molybdate tetrahydrate (0.404 mmol) and thiourea (6.56 mmol). The soaked reaction mixture was placed inside a hydrothermal reactor and incubated for 18 hours at 195 °C in a hot air oven (reaction (1)). After incubation, the hydrothermal reactor was allowed to cool to ambient temperature. The *in situ* grown DRH-MoS_2_ was repeatedly washed with Millipore water until the filtrate colour turned transparent. The produced DRH-MoS_2_ was further dried at 60 °C in a hot air oven and utilised for the dye remediation as discussed.

1 2(NH_4_)_6_Mo_7_O_24_·4H_2_O + 28SC(NH_2_)_2_ + 22H_2_O → 14MoS_2_ + 28CO_2_ + 7O_2_

### 2.3 Adsorption of MG on DRH

A stock solution of 3000 mg L^−1^ was prepared from powdered MG dye in distilled water. Different working solutions of concentration 100 mg L^−1^, 200 mg L^−1^, 400 mg L^−1^, 600 mg L^−1^, 1000 mg L^−1^, and 2700 mg L^−1^ were prepared from the stock solution. 0.2 g of DRH was mixed into 50 mL of the prepared stocks, and dye adsorption experiments were performed for 4 hours. The effects of pH and temperature on the adsorption behaviour of the MG on DRH were studied for pH (3, 5, 7, 9 and 11), and temperatures (*T* = 30, 35, 40, 45, and 50 °C) on the adsorption behaviour of the MG on DRH were studied in detail. The absorption of MG dye was taken at 617 nm using an Agilent spectrophotometer throughout the study. The percentage adsorption of the MG was calculated from eqn (2).2
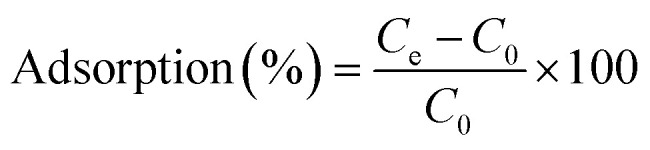
where *C*_e_ (mg L^−1^) is the MG concentration at equilibrium, and *C*_0_ is the initial concentration of MG.

### 2.4 Photodegradation of MG in dark and light using DRH-MoS_2_

DRH-MoS_2_ (0.2 g) was used to carry out the photodegradation of MG (at different concentrations of 100 mg L^−1^, 200 mg L^−1^, 400 mg L^−1^, 600 mg L^−1^, 1000 mg L^−1^, and 2700 mg L^−1^) with working volume of 50 mL under white light source (intensity-21.6 W m^−2^, Ocean slim LED panel 20 W) and dark condition for 4 hours. The effects of temperature (*T* = 30, 35, 40, 45, and 50 °C) and pH (3, 5, 7, 9 and 11) on the photodegradation behaviour were studied in detail. The reaction vessels for the photodegradation reaction were kept at a distance of 15 cm from the light source throughout the study for all the experiments performed. The eqn (3) was used for the calculation of percent degradation:3
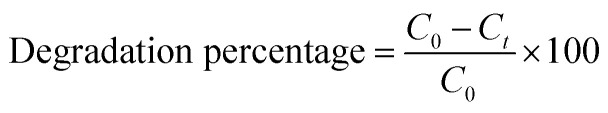
*C*_0_ and *C*_*t*_ are the initial concentration of MG and MG concentration at any time ‘*t*’.

#### 2.4.1 Continuous photodegradation and reusability of the DRH-MoS_2_

Continuous photodegradation of the MG (dye concentration = 1200 mg L^−1^) was performed in a plug flow reactor (dimension, *L* = 9 cm, and *D* = 2 cm) embedded with 0.5 g DRH-MoS_2_ catalysts. The continuous degradation process was carried out for five cycles, each consisting of 4 hours of dye degradation using 300 mL of MG dye solution. The experiments were performed under a light source with an intensity of 21.6 W m^−2^ at a distance of 15 cm, and peristaltic pumps were used for the continuous flow of the dye (rate = 5 mL min^−1^). The reusability of the DRH-MoS_2_ catalyst was also checked using the MG dye concentration of 300 mg L^−1^ for five repetitive cycles.

### 2.5 Antibacterial properties of DRH-MoS_2_

The antibacterial properties of the DRH-MoS_2_ were analysed with Gram-positive bacteria *S. aureus* and Gram-negative bacteria *E. coli* on a nutrient agar (NA) plate. *S. aureus* and *E. coli* were cultured on NA plates. Subsequently, a suspension of sonicated DRH-MoS_2_ was carefully spread on both plates, followed by incubation in an incubator shaker at 37 °C for 18 hours. The diameter of the zone formed after the incubation was checked and reported.

## 3. Results and discussion

**Fig. 1:**
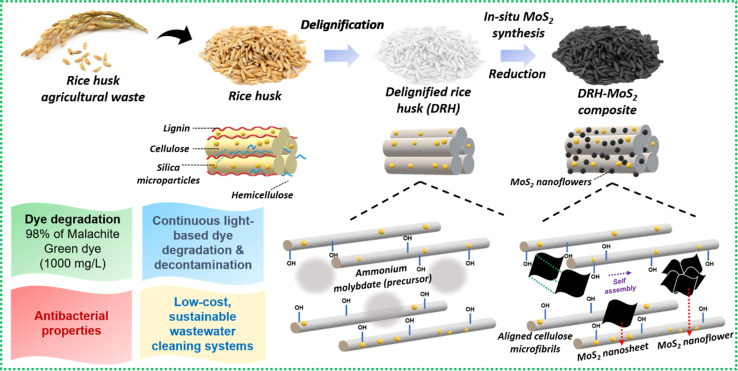
Schematic for chemical modification of RH through strategic delignification followed by *in situ* growth of MoS_2_ nanosheets, which undergoes self-assembly to form MoS_2_ nanoflowers with capabilities for the simultaneous dye adsorption, degradation, and microbial decontamination.

Rice, one of the most consumed crops globally, generates large amounts of waste in the form of straws or husks during its processing, accumulating and posing harmful effects to the environment and aquatic life when disposed of conventionally (burning or landfilling).^39^ Therefore, the current investigation valorises the abundantly available, low-cost, lignocellulosic RH waste to remove chemo-biological contaminants from wastewater by simultaneous photodegradation of organic dyes and decontaminating pathogenic microbiota. Delignification of RH removes the aromatic lignin, exposing the cellulosic backbone's hydroxyl groups and providing sites for *in situ* growth of MoS_2_ nanosheets within the aligned cellulosic microstructure.^40^ MoS_2_ nanosheets localise randomly in the interlayers of cellulose microfibers self-assembling to form a nanoflower-like architecture as schematically shown in Fig. 1. The performance of DRH-MoS_2_ for adsorption and photodegradation of MG dye, a known carcinogenic agent mostly found in wastewater of the textile and food industry, to non-toxic forms^41^ was evaluated through mechanistic and kinetic studies. MoS_2_, due to its catalytic properties, can absorb light in the visible range and generate free radicals that can potentially degrade organic contaminants such as dyes. The degradation efficiency of MG was evaluated under various temperatures, pH, and concentrations for dye adsorption and photodegradation. ROS generation by DRH-MoS_2_ can also inflict damage to pathogenic microbes by cell lysis, which was evaluated through antibacterial studies. The improved MG removal efficiency and recyclability of DRH-MoS_2_ during continuous degradation in plug-flow reactors show potential for industrially scalable and sustainable wastewater purification technologies derived from agro-biomass residues.

### 3.1 Physicochemical and functional properties of DRH and DRH-MoS_2_ formation

**Fig. 2:**
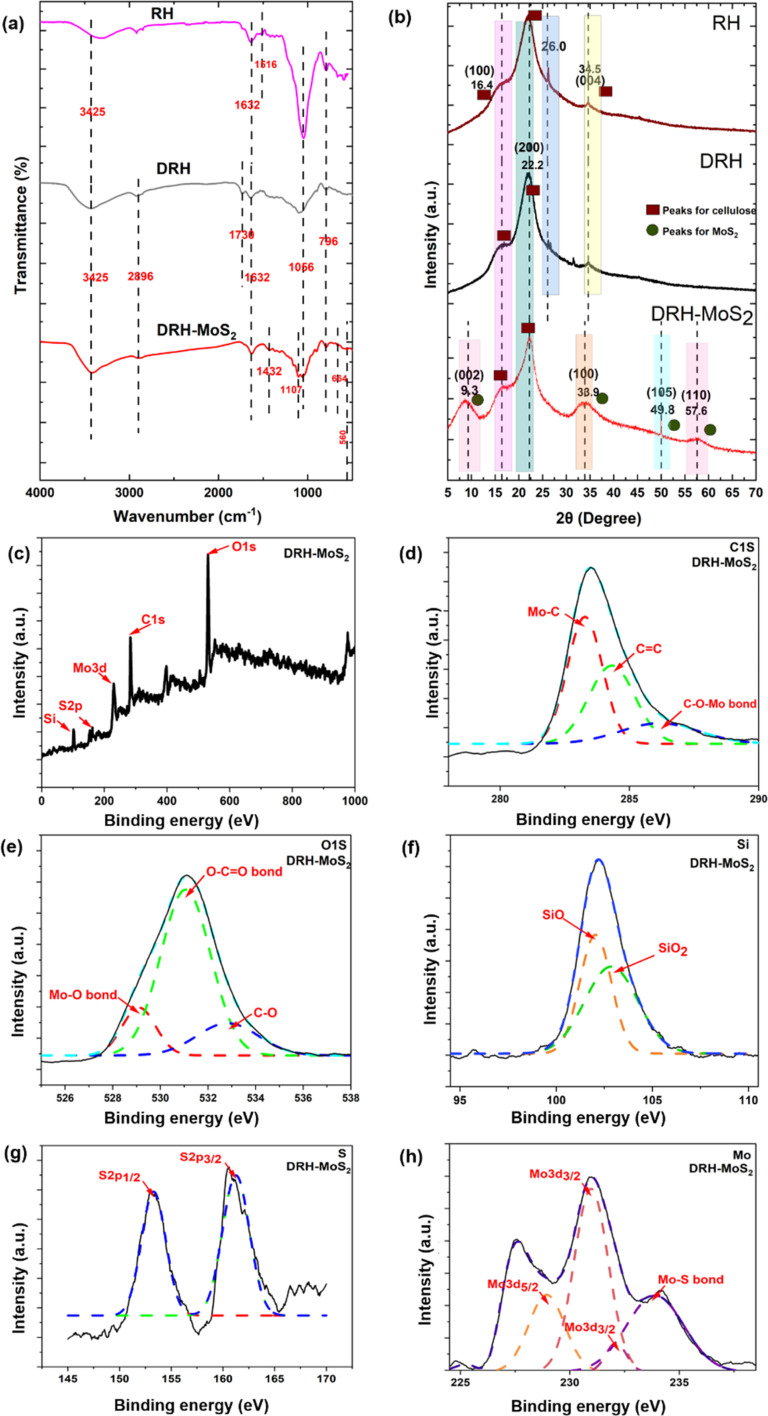
Physicochemical, functional, and structural properties: (a) FTIR spectrum, (b) XRD spectrum of RH, DRH, and DRH-MoS_2_, (c) XPS survey a broad spectrum of DRH-MoS_2_, with small scan section of (d) C1s, (e) O1s, (f) Si2p, (g) S2p and (h) Mo3d of DRH-MoS_2_.

The ATR-FTIR spectroscopy was used to determine functional groups in RH and DRH and confirm the *in situ* growth of MoS_2_ nanosheets in DRH-MoS_2_ samples (Fig. 2(a) and Table S7†). The availability of IR peaks at 796 cm^−1^, 1066 cm^−1^, 1632 cm^−1^, and 3425 cm^−1^ in RH, DRH, and DRH-MoS_2_ samples correspond to Si–O bond, asymmetric Si–O–Si stretching vibrations, C=O stretching vibration of the carbonyl group in aldehyde and ketones, and bending vibration of –OH respectively. IR spectra at 2896 in DRH and DRH-MoS_2_ correspond to the C–H stretching of cellulose achieved after the delignification of RH. The availability of peak at 1616 cm^−1^ in RH and 1736 cm^−1^ in DRH sample corresponds to the C=C bond of aromatic carbon of lignin and C=O stretching of acetyl groups arises due to the presence of hemicellulose respectively. Lastly, the IR peaks at 560, 664 cm^−1^, 1107 cm^−1^ and 1432 cm^−1^ in the DRH-MoS_2_ sample correspond to Mo–S,^42^ O–Mo–O stretching^43^ S–S bond, and aliphatic C–H stretching respectively. The peaks at 560, 664 cm^−1^, and 1107 cm^−1^ confirms the *in situ* growth of MoS_2_ in the inter-layers of cellulose in DRH and further confirmed by XPS and EDS studies, as discussed in the subsequent section.

XPS studies of DRH and DRH-MoS_2_ were conducted to analyse the changes in the chemical linkages during the *in situ* growth of MoS_2_ on the DRH surface. Fig. S1(a)† represents the XPS survey of DRH, with peaks at 533.08 eV, 285.08 eV, and 103.08 eV representing O1s, C1s, and Si bonds, respectively. In the high-resolution spectrum C1s in Fig. S1(b),† peaks at 284.4 eV, 286.02 eV, and 287.8 eV represent C–H, C–O–C, and C=O bonds, respectively, which represent the linkages from cellulose (DRH). In Fig. S1(c),† the peaks at 533.4 eV,531.0 eV and 532.4 eV in the broad spectrum of O1s correspond to the C–OH bond, SiO_2_ and C=O bond arises due to the presence of inherent silica in RH.^44^ In Fig. S1(d),† narrow scanning of Si presents a peak at 103.2 eV that could be further deconvoluted to SiO_2_ (103.8 eV) and Si–O–Si (103.1 eV) and Si–C (101.2 eV),^45^ occurring due to silica embedded in aligned cellulose microfibrils. No peaks were observed in the XPS of DRH for the Mo and S, as shown in Fig. S1(e) and (f),† with only noise observed in the spectra. The wide range XPS survey of DRH-MoS_2_ (Fig. 2(c)) presents a peak for O1s, C1s, Mo3d, S2p, and Si groups at binding energy 531.1 eV, 283.6 eV, 231 eV, 153 eV, and 102.3 eV respectively. The narrow scan of C1s (depicted in Fig. 2(d)) revealed three distinct subpeaks at 283.3, 284.4, and 285.6 eV, corresponding to the Mo–C bond, C=C, and Mo–O–C bond, respectively. Following the *in situ* growth of MoS_2_ on DRH, the emergence of Mo–C and Mo–O–C bonds indicates the formation of covalent bonds between MoS_2_ and cellulose within DRH.^46^ The deconvoluted Mo3d peaks (Fig. 2(h)) show Mo–S bonds (233.9 eV), Mo3d_5/2_ (228.9 eV) and Mo3d_3/2_ (231 eV and 232.1 eV), signifying the Mo(iv) oxidation state of MoS_2_.^33^ The narrow scanning of Si (Fig. 2(f)) presents a peak of 102.3 eV that can be further deconvoluted to SiO_2_ (102.1 eV) and Si–O (102.9 eV).^47^ The peaks for S2p (Fig. 2(g)) were observed at 153.2 eV and 161.3 eV for S2p_1/2_ and S2p_3/2_, attributed to the binding energy of S^2−^.^33^ The narrow O1s spectrum in Fig. 2(e) was deconvoluted, revealing three subpeaks at binding energies of 531.1 eV, 532.7 eV, and 529.1 eV representing O–C=O bond, C–O bond and Mo–O bond respectively.^48–50^ The in-depth analysis of XPS and FTIR spectroscopy suggests that molybdenum salts adsorbed onto hydroxyl groups in DRH through electrostatic interactions underwent successful reduction to form MoS_2_ nanosheets. Moreover, it was observed that MoS_2_ nanosheets dispersed uniformly in DRH underwent self-assembly to form nanoflower structures, which were governed by hydrogen-bonded interactions (Fig. 1). The elemental composition of DRH and DRH-MoS_2_ (Table S1†) shows the presence of S ∼ 3.36 and Mo ∼ 1.05 atomic %, which were absent in the DRH and in line with EDX spectroscopy studies. To further confirm morphology attributes of *in situ* grown MoS_2_ on the surface of DRH, SEM investigations were also performed, as discussed subsequently. Additionally, the energy band gap of DRH-MoS_2_ was assessed through UV-DRS analysis. As depicted in Fig. S2,† Tauc's plot revealed a determined band gap of 1.37 eV for DRH-MoS_2_. The observed narrow band gap in DRH-MoS_2_ further suggests the potential of these composites to serve as effective photocatalysts, particularly under visible light exposure.^36^

### 3.2 Structural investigation of DRH and DRH-MoS_2_

XRD diffractogram helps understand the influence of hydrothermal treatment on the *in situ* growth of MoS_2_ nanosheets on the cellulosic backbone in DRH. In the XRD diffractogram of RH, DRH and DRH-MoS_2_ three distinct peaks were observed at 2*θ* = 16.4°, 22.2° and 34.5° (Fig. 2(b)), which corresponds to the (110), (200), (004) crystal planes of cellulose I allomorphs, respectively. The sharp peak at 2*θ* = 26.0° in DRH and RH samples were primarily due to presence of silica.^51^ The diffraction pattern remained consistent throughout the cellulose extraction process. Still, there was a noticeable increase in peak intensities in DRH after delignification, resulting in higher crystallinity values for each sample. RH's crystallinity index (C.I.) was found to be 45.6%, which, on delignification, improved to 51.3% in DRH. However, after hydrothermal treatment, XRD spectra of DRH-MoS_2_ show three new and distinct peaks at 2*θ* = 9.3°, 33.9°, 49.8° and 57.6° which represent lattice planes (002), (100), (105) and (110) respectively correspond to the hexagonal 2H-MoS_2_ phase (Fig. 2(b)).^40^ It was observed that after the *in situ* growth of MoS_2_ nanosheets, the crystallinity index (C.I.) of DRH-MoS_2_ reduced to 30% compared to the C.I. of DRH ∼ 51.3%. The reduction in C.I. for DRH-MoS_2_ could be attributed to the hydrolysis, and degradation of cellulose structure on prolonged reaction time at high temperatures during hydrothermal reaction. Following hydrothermal treatment, the peaks of DRH-MoS_2_ exhibit broadening, accompanied by a decrease in peak intensity, leading to a reduction in crystallinity.^52^ The change in the crystallite size and *d*-spacing were also observed in both RH and DRH samples post-hydrothermal treatment (as discussed in Table S2†).

### 3.3 Morphological properties of DRH and DRH-MoS_2_

**Fig. 3:**
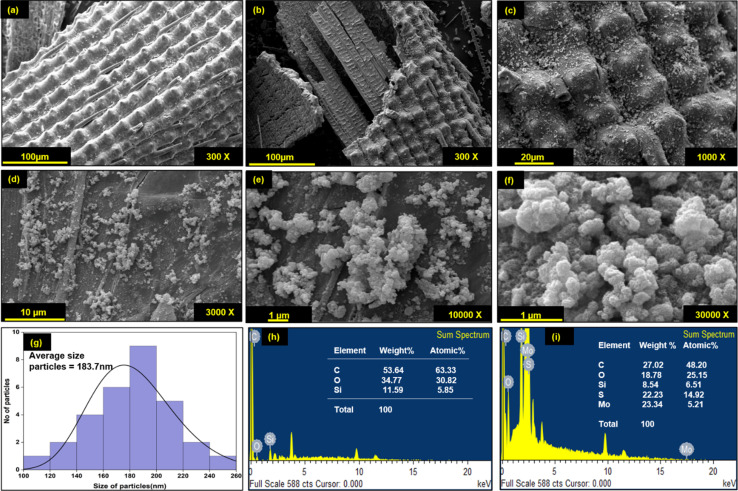
SEM-micrograph of (a) DRH and (b) DRH-MoS_2_, (c) DRH-MoS_2_ at higher magnification (1000×), dispersed MoS_2_ nanoflower on DRH (d) at 3000×, (e) 10 000× and (f) 30 000× magnification, (g) average particle size distribution of MoS_2_ nanoflowers, EDS spectrograph of (h) DRH, (i) DRH-MoS_2_.

Morphological changes in DRH during *in situ* growth of MoS_2_ and their distribution were determined by SEM-EDXS analysis. As shown in Fig. 3(a), DRH shows an aligned, porous, and rolled shape structure with regularly spaced silica microparticles. The surface of DRH was observed to have a protruded and rough appearance due to the presence of silica at the outer surface (Fig. 3(b)), as also reported in earlier studies.^53^ The EDXS analysis of DRH confirms the presence of Si with 11.56 w%, along with other constituents C ∼ 53.64 w% and O ∼ 34.77 w% (Fig. 3(f)). The low magnification SEM micrograph of DRH-MoS_2_ shows the distribution MoS_2_ on DRH which, on high magnification (Fig. 3(d)). The formation of MoS_2_ nanoflower structures results from the self-assembly of MoS_2_ nanosheets formed during *in situ* synthesis, originating from hydrogen-bonded interactions, as discussed in the previous section. EDXS analysis of DRH-MoS_2_ confirms the presence of Mo with ∼23.34 w%, with other constituents such as C ∼ 27.02 w%, O ∼ 18.78 w%, S ∼ 22.23 w%, and inherently presence of Si ∼ 8.54 w%, Fig. 3(g) which is in-line with the XPS studies. On pictorial investigations (Fig. 3(h)–(j)), it was observed that RH shows a pale-yellow colour which, on delignification DRH, appears to be white and, on hydrothermal treatment, forms a black colour appearance due to formation of MoS_2_. Therefore, microscopic analysis of DRH-MoS_2_ confirms the growth, uniform distribution, and self-assembly of MoS_2_ nanosheets in cellulose interlayers, making it suitable for pollutant dye remediation, as discussed in subsequent sections.

### 3.4 Adsorption of MG on DRH

**Fig. 4:**
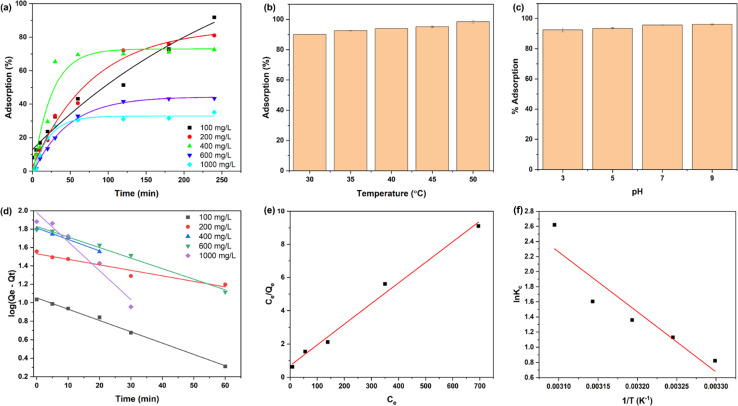
Adsorption of MG: (a) % adsorption of MG at various concentrations on DRH at various time intervals, (b) effect of different temperatures, (c) pH on % adsorption of MG (d) linear fitting of pseudo-first-order kinetic model, (e) Langmuir isotherm and (f) studies of adsorption thermodynamic parameters.

Fig. 4(a) shows the adsorption behaviour of DRH at various MG concentrations (100, 200, 400, 600, and 1000 mg L^−1^). The adsorption of MG onto the DRH surface increases steadily over 120 minutes, reaching a plateau of 200 min with a maximum adsorption capacity of 88 mg g^−1^. The highest adsorption percentage was 91% at 100 mg L^−1^ but dropped to 35.2% at a higher MG concentration of 1000 mg L^−1^. At first, there were unoccupied binding sites for dye molecule attachment, but as they became saturated, the already-filled binding sites could potentially repel incoming ions. Additionally, alterations in the concentration gradient between the solid surface and the solution might have occurred or, due to the presence of available binding sites, led to increased competition among ions, resulting in a stagnation of the adsorption process. Previous research has also reported similar findings, indicating that the adsorption process may decline over time as the binding sites become saturated.

#### 3.4.1 Effect of temperature and pH

The effect of temperature on the dye adsorption capacity of DRH was investigated at five different temperatures (30, 35, 40, 45, and 50 °C), as shown in Fig. 4(b). The percentage of MG adsorption increased consistently with temperature, reaching a maximum of 98.5% at 50 °C, indicating that the adsorption phenomenon is endothermic. The slight increase in the percentage of MG degradation can be accredited to the increased kinetic energy caused by the temperature increase, which improves the mobility and diffusion of dyes to the surface of the adsorbent. This, in turn, promotes increased MG adsorption on the surface of DRH.

Fig. 4(c) depicts the effect of pH (3, 5, 7, and 9) on MG adsorption on the surface of DRH. At lower pH values, MG adsorption percent was relatively lower, with maximum adsorption (96.2%) occurring at pH 9. The p*K*_a_ of MG is 4.52, indicating that dye molecules exist predominantly in their cationic form when the pH of the dye is lower than p*K*_a_. The decreased adsorption at pH 3 is due to the protonation of H^+^ ions in the solution due to which abundancy of H^+^ ions increase on the DRH surface. Therefore, the positive charge on MG and H^+^ ions present on DRH surface causes electrostatic repulsion, preventing adsorption. However, as pH rises, the availability of OH^−^ (deprotonated) on DRH surface rises, resulting in a decrease in electrostatic repulsion, results in improved interaction between MG and DRH surface, thus higher adsorption.

#### 3.4.2 MG adsorption kinetics on DRH

Two kinetic models, such as the pseudo-first-order and pseudo-second-order kinetic models, were fitted to examine the behaviour of adsorption and rate of MG adsorption on the surface of DRH. Fig. 4(d) shows linear fitting of the pseudo-first-order kinetics and their parameters mentioned in Table S3.† The correlation coefficient (*R*^2^) values were calculated to be ∼0.94–0.99, indicating that the experimental data best fit the pseudo-first-order, representing that the MG adsorption on DRH is directly proportional to the number of active sites available. This result suggests that the adsorption of MG on the surface of DRH was predominantly governed by physisorption. Similar results were also observed for adsorption of MG on neem leaves.^54^

#### 3.4.3 Adsorption isotherm

To explore the adsorption capacity and the interaction between MG and DRH-MoS_2_, two widely recognised isotherm models, namely Langmuir (Fig. 4(e)) and Freundlich, were examined. These isotherm models provide insights into the distribution of adsorbate on solid adsorbent when equilibrium is reached between the liquid and solid phases of the medium. The experimental adsorption data were fitted to both models and based on the correlation coefficient (*R*^2^) values obtained, the Langmuir model (*R*^2^ −0.98) is well-suited for studying the removal of dyes using DRH in this study. This suggests that the adsorption sites on DRH are evenly distributed, and the adsorption process takes place uniformly. The monolayer adsorption capacity (*Q*_o_) was 80.45 mg g^−1^ for the Langmuir isotherm. The value 1/*n* for the Freundlich isotherm was 0.4, less than 1, suggesting that the adsorption phenomenon could have been more cooperative. The value of *n* ∼ 2.5 observed was more significant than one, suggesting that the adsorption of MG on DRH is strongly affected by physical adsorption, indicating a favourable influence, as per the Freundlich isotherm.

#### 3.4.4 Adsorption thermodynamics

The thermodynamic parameters such as enthalpy (Δ*H*^o^), entropy (Δ*S*^o^), and Gibb's free energy (Δ*G*^o^) were calculated to characterise the adsorption process. A plot of the natural logarithm of the equilibrium constant (ln *K*_c_) against the inverse of temperature (1/*T*) was used to calculate these values (Fig. 4(h)). The slope of the plot was used in determining the value of Δ*H*^o^, whereas the intercept provided the value of Δ*S*^o^. The parameter values mentioned in Table S5† show that as the temperature rises from 30 to 50 °C, Δ*G*^o^ values for MG adsorption on DRH decrease from −2.16 to −10.58 kJ mol^−1^. This decrease suggests MG adsorption on DRH becomes more favourable and spontaneous at higher temperatures.^55^ The positive value of Δ*H*^o^ (65.89 kJ mol^−1^) indicates that MG adsorption on DRH is endothermic, requiring energy input for improved adsorption efficiency.^56^ Furthermore, a low entropy change (Δ*S*^o^) value (0.22 kJ K^−1^ mol^−1^) indicates that DRH has a high affinity for MG with a slight rise in randomness at the interface between DRH and dye solution. However, due to poor adsorption capacity, DRH was chemically modified with MoS_2_*via* an *in situ* hydrothermal process capable of degrading the adsorbed dye available in bulk.

### 3.5 Photodegradation of MG under light and adsorption under dark conditions by DRH-MoS_2_

**Fig. 5:**
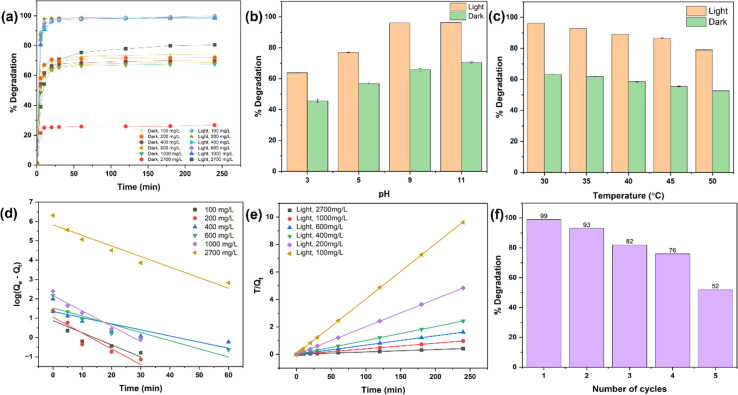
Photodegradation of MG by DRH-MoS_2_: (a) % degradation of MG at various concentrations by DRH-MoS_2_ in light and dark, (b) effect of pH and, (c) temperature on % degradation of MG, (d) linear fitting of pseudo-first-order, and (e) pseudo-second-order kinetic model, and (f) cyclic photodegradation and reusability of DRH-MoS_2_.

Fig. 5(a) shows the catalytic degradation of MG dye at different initial concentrations under both light and dark conditions using a fixed amount of DRH-MoS_2_. The degradation of MG showed excellent results under light conditions with a removal efficiency of 99% for dye concentration from 100–1000 mg L^−1^, which reduced to ∼84.7% at a very high MG concentration of 2700 mg L^−1^. The degradation rate was fast within the first five minutes for 100 to 1000 mg L^−1^, with degradation efficiency reaching ∼88–90%. Faster photodegradation of MG using *in situ* grown DRH-MoS_2_ might be due to the presence of porous delignified cellulose structures (DRH) and the presence of MoS_2_ nanoflower, which provides sites for both adsorption of dye and catalytic degradation.^57^ At first, MG is adsorbed onto the DRH-MoS_2_ structures, aiding in the removal of the dye. Subsequently, the photocatalytic properties of MoS_2_ lead to an improved overall performance.^58^ The enhancement of photodegradation by DRH-MoS_2_ under light conditions can be attributed to the presence of MoS_2_ in the optical band gaps, which facilitate the generation of additional electrons and holes when exposed to light.^23^ When DRH-MoS_2_ was subjected to light irradiation, electrons were excited from the valence band to the conduction band, forming holes in the valence band.^59–61^ These photo-generated carriers rapidly recombine to form electron–hole pairs, and their presence on water or oxygen molecules causes the formation of reactive oxygen species (ROS), such as hydroxyl radicals or superoxide radicals. These ROS are critical in degrading organic dyes such as MG by breaking them down into smaller, colourless, and non-toxic molecules. Moreover, the rough surface of DRH and the high volume-to-surface area of MoS_2_ nanoflowers provide more sites of interaction with photons, resulting in the generation of a large number of ROS. The ROS generated interacts with dye adsorbed onto the cellulose surface in DRH-MoS_2_ and the MG dye solution, enhancing its degradation.

Under dark conditions, adsorption of MG shows reduced efficiency ranging from ∼74 to 68% when tested with dye concentrations of 100 to 1000 mg L^−1^. On further increasing at a very high dye concentration to 2700 mg L^−1^, only 26.6% adsorption was achieved. The decrease in adsorption efficiency on increasing MG concentration might be due to the absence of ROS reactive species involved in the degradation phenomenon. Therefore, it could be observed that DRH-MoS_2_ shows improved photodegradation (by 25–30%) with the ability to remove dye even at a high MG concentration of 2700 mg L^−1^, which is typically several orders of magnitude higher than dye levels found in standard industrial wastewater.

#### 3.5.1 Effect of pH and temperature on MG degradation

pH strongly influences dye degradation because it causes protonation or deprotonation with alteration in the surface charge of dye molecules, thereby changing the interaction with the adsorbate. As shown in Fig. 5(b), the degradation of MG increased with an increase in the pH of the dye solution in both light and dark conditions. The degradation of MG in the presence of light ranged from 63% to 93% across pH values ranging from 3 to 11. However, even in the absence of light, degradation increased from 45% to 70% as the pH increased from 3 to 11, albeit at a significantly lower rate than in the presence of light. The catalyst surface becomes protonated at lower pH levels, and MG, a cationic dye, causes electrostatic repulsion, resulting in reduced degradation.^62^ As presented in Fig. 5(c), the degradation of MG (at 300 mg L^−1^) decreases by increasing the temperature in both light and dark conditions. The degradation of MG decreased from 96 to 78% under light conditions in the temperature range of 30–50 °C. Under the dark conditions, the percentage degradation of MG reduced from 63.3% to 52.5% in the temperature range of 30–50 °C. The decrease in % degradation of MG on increasing temperature might be due to a change in the solubility of MG or increased system kinetic energy. The improved kinetic energy of MG molecules may result in their removal from the surface of DRH-MoS_2_ without being subjected to adsorption, followed by photodegradation.^63^

#### 3.5.2 Kinetic study

The pseudo-first-order and pseudo-second-order kinetic models were studied to understand the mechanism and adsorption behaviour of MG on DRH-MoS_2_ (Fig. 5(d) and (e)). The pseudo-second-order kinetic model fitted well with MG photodegradation experimental data, showing good linear behaviour with a coefficient of correlation *R*^2^ ∼ 0.99. The kinetic parameters for pseudo-second-order and pseudo-first-order kinetic models are shown in Table S6.† As per pseudo-second-order kinetics, the adsorption-*cum*-photodegradation of MG dye onto the DRH-MoS_2_ is controlled by chemisorption. This chemisorption is determined by exchanging or sharing electrons between MG and DRH-MoS_2_. The kinetic rate constant for pseudo-second-order kinetic models increased with MG concentration, suggesting that MG molecules adsorbed on the surface of the DRH-MoS_2_ result in a faster adsorption rate, which is in line with the experimental studies. The prevalence of such forces with an improved rate of MG dye adsorption onto DRH-MoS_2_, even at high dye concentrations (∼2700 mg L^−1^), led to improved degradation efficiency, making it suitable for industrial-scale wastewater remediation technologies.

### 3.6 Continuous photodegradation of MG with DRH-MoS_2_ and its reusability

**Fig. 6:**
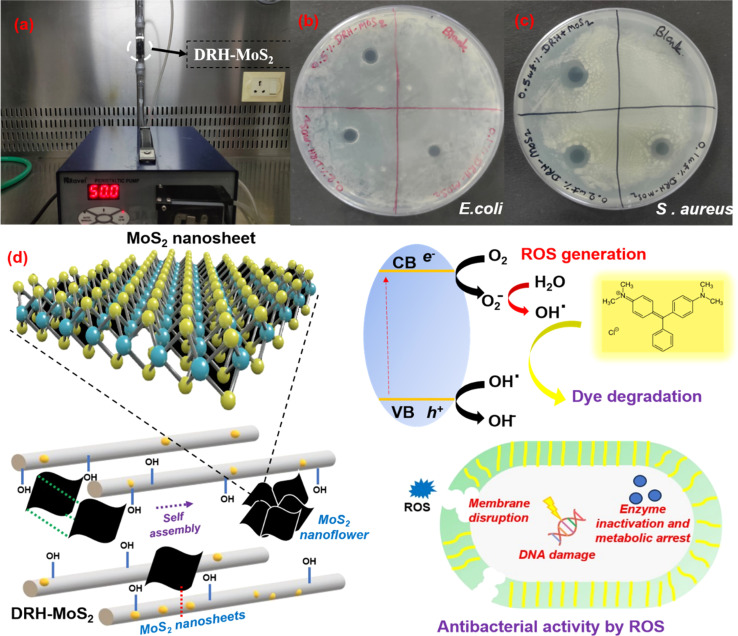
Continuous photodegradation of MG and microbial decontamination: (a) plug flow reactor for the continuous photodegradation of MG, (b) antibacterial activities of DRH-MoS_2_ against *E. coli*, (c) antibacterial activities of DRH-MoS_2_ against *S. aureus*, (d) proposed mechanism for continuous dye degradation and antibacterial properties of DRH-MoS_2_.

A continuous system with dye degradation capabilities makes it easily scalable and translational at an industrial scale with wastewater processing capabilities at higher volume and throughput. The continuous photodegradation experiment using a plug flow reactor (as shown in Fig. 6(a)) was carried out for five cycles, and each cycle continued for 4 hours with 300 mL of MG, illuminated using a 20 W white light source placed at a distance of 15 cm. The maximum degradation of 99.9% MG was observed in the first cycle, which reduced to 93% in the second cycle and 82% and 76% in the third and fourth cycles, respectively (Fig. 5(f)). These results show that DRH-MoS_2_ could continuously degrade 600 mL of MG with more than 90% efficiency. However, the adsorption capacity of DRH-MoS_2_ reduced to 52% after the fifth cycle, possibly due to a reduction in catalytic activity and fewer sites available for the adsorption of MG. This challenge can be overcome by the regeneration of DRH-MoS_2_ to rejuvenate the catalytically active sites or by increasing the amount of catalyst bed used in the plug flow reactor (only 0.5 g was used in the present study). The above results conclude that DRH-MoS_2_ could be ideally used for continuous removal of pollutant dye with highly effective degradation capabilities for five cycles and microbial decontamination capabilities evaluated in subsequent sections.

### 3.7 Free radical scavenging

DRH-MoS_2_ in presence of light shows strong photocatalytic activity and DPPH-based antioxidant activity tests were carried out to confirm the ROS formation which are required for improved dye photodegradation and microbial decontamination. The DRH-MoS_2_ showed DPPH scavenging activity in the range of 43.5% to 74.2% for the concentration ranging from 0.05 to 0.25 mg L^−1^ (Table S9†). These results confirms that photocatalytic activity of DRH-MoS_2_ through free radical scavenging activity which enhances with increased concentration.^64,65^

### 3.8 Antibacterial properties of DRH-MoS_2_

The antibacterial properties of DRH-MoS_2_ were studied on *S. aureus* (Gram-positive bacteria) and *E. coli* (Gram-negative bacteria). The suspension of DRH-MoS_2_ was gently placed on the freshly cultured plates of *E. coli* and *S. aureus* using three different DRH-MoS_2_ concentrations (0.1, 0.2, and 0.5 wt%). The inhibition zones for the *E. coli* cultured plate were 7, 8, and 8 mm, which slightly increased upon an increase in the concentration of composites. However, in the case of *S. aureus* cultured plate, the inhibition zones were 12, 12, and 13 mm. The generation of inhibition on both the cultured plates confirms the antibacterial properties of the developed DRH-MoS_2_. The antibacterial property of DRH-MoS_2_ might be due to the presence of inherent photocatalytic properties of MoS_2_. When MoS_2_ is directly exposed to light sources, ROS are generated, which aid in destroying or inactivating the microorganisms by the cell membrane lysis.^66^ The membrane disruption causes phospholipid extraction and harms the structural integrity of bacteria's lipid membrane. Therefore, the present study provides a unique strategy for RH valorisation through *in situ* grown MoS_2_ for simultaneous dye adsorption, photodegradation and microbial decontamination.

## 4. Conclusion

In conclusion, this study successfully transformed RH into a biocatalyst through strategic delignification and *in situ* growth of MoS_2_*via* the hydrothermal method. The resulting DRH-MoS_2_ nanosheets demonstrates 2H-MoS_2_ phase structure with outstanding catalytic performance, possessing a band gap measuring 1.37 eV, and exhibits an impressive scavenging activity of 74.2%. The developed composites showed excellent photodegradation efficiency up to 500 mg L^−1^ dye concentration. DRH shows adsorption capacity of 88.03 mg g^−1^ which improves on growth of DRH-MoS_2_ to 181.16 mg g^−1^ under dark conditions and further enhances to 550 mg g^−1^ under light exposure. Continuous photodegradation with recycling confirmed the composite's reusability for three cycles with over 80% degradation capacity. Importantly, DRH-MoS_2_ also exhibited robust antibacterial activity against *S. aureus* and *E. coli*. Overall, these findings highlight the potential of DRH-MoS_2_ as a versatile solution for pollutant dye adsorption, photodegradation and pathogen removal in wastewater, showcasing a sustainable and scalable valorization approach for abundant rice-biomass waste.

## Author contributions

Rahul Ranjan: conceptualization, investigation, methodology, formal analysis visualization, writing – original draft: investigation, validation, writing – review editing. Smruti B. Bhatt: writing an original draft, data curation, Rohit Rai: conceptualization, writing – review & editing, Sanju Kumari Sharma: writing – review & editing, Rishabh Ranjan: formal analysis, and methodology, Ankit Bharti: writing – review & editing Prodyut Dhar: supervision, conceptualization, validation, project administration, funding acquisition. All contributors collectively authored the manuscript, and all authors have approved the final version.

## Conflicts of interest

There are no conflicts to declare.

## Supplementary Material

RA-014-D4RA00862F-s001
