# Supplementary material for: Rice husk valorisation by in situ grown MoS2 nanoflowers: a dual-action catalyst for pollutant dye remediation and microbial decontamination
Source: RSC Adv. 2024 Apr 16;14(17):12192–203. doi: 10.1039/d4ra00862f (PMC11019664; doi:10.1039/d4ra00862f)
Supplement: RA-014-D4RA00862F-s001 [file RA-014-D4RA00862F-s001.pdf]

Electronic Supplementary Material (ESI) for RSC Advances.
This journal is © The Royal Society of Chemistry 2024

Supplementary information

Rice husk valorisation by *in-situ* grown MoS_2 nanoflowers: A dual-action catalyst for pollutant dye remediation and microbial decontamination

*Rahul Ranjan[1], Smruti B Bhatt[1], Rohit Rai[1], Sanju Kumari Sharma[1], Rishabh Ranjan[2], Ankit Bharti[1] and Prodyut Dhar[1]**

[1] School of Biochemical Engineering, Indian Institute of Technology (BHU), Varanasi, Uttar Pradesh-221005, India

[2] Department of Biotechnology, National Institute of Technology Durgapur, West Bengal 713209

**Corresponding author e-mail:* prodyut.bce@iitbhu.ac.in

Material characterisation

Fourier transformation infrared spectroscopy (FTIR)

The oven-dried RH, DRH, and DRH-MoS_2 samples were analysed using FTIR spectroscopy in Attenuated Total Reflection mode. The analysis used a Nicolet iS5 instrument from THERMO Scientific US in the 500-4000 cm-1 range. The samples were gently placed directly onto the diamond crystal, pressed using a knob, and scanned 64 times, with the spectra recorded at a resolution of 4 cm^{-1}.

X-ray photoelectron spectroscopy (XPS)

The chemical composition of DRH-MoS_2 and DRH was analysed using a PHI5000 Versaprobe III scanning XPS microprobe instrument. The sample was initially fixed on a holder before being placed inside the analysis chamber. Narrow scans had a step size of 0.1 eV, while survey scans had a step size of 1 eV. The investigation used an $Al\text{-}k_{\alpha}$ anode, a 400 m beam spot size, and a binding energy range of -10 eV to 1350 eV. The experiment was conducted under vacuum, with pressures ranging from 10^{-8} to 10^{-9} mbar. The obtained spectra were analysed

using MultiPak 9.9 version (ULVAC-PHI), and deconvolution of the peaks was carried out using Origin software (2023b).

X-ray diffraction (XRD)

The synthesised composite DRH-MoS_2 and DRH powder was subjected to XRD analysis using MiniFlex 600 (Bench Top XRD instrument, Rigaku, Japan). The powdered samples were then evenly distributed onto aluminium holders with a flat glass spreader. Cu-k_α radiation is generated from a copper anticathode used for the analysis and operated at 40 kV voltage and 20 mA current. Scanning was done from 2 (θ) angles ranging from 5° to 90° at a scan rate of 5° per minute and a step size of 0.02. The *Segal equation* (eq.1), *Bragg's equation* (eq.2), and *Scherrer equation* (eq.3) were used to calculate the crystallinity index (*CI*) and the distance between crystal planes (*d*-spacing) and crystal size (*D*), respectively.

$$CI = \frac{I_{002} - I_{am}}{I_{002}} \quad \text{(eq.1)}$$

$$n\lambda = 2dsin\theta \quad \text{(eq.2)}$$

$$D = \frac{k\lambda}{\beta\ Cos\theta} \quad \text{(eq.3)}$$

diffraction peak I_{002} corresponds to the crystalline plane, while the intensity peak I_{am} represents the peak for the amorphous region at an angle of 2θ around 18.6°. The symbol λ represents the wavelength of Cu K_α radiation, and n signifies the number of wavelengths. The parameter d refers to the spacing between two adjacent layers of atoms.

Scanning electron microscopy (SEM)

A Carl Zeiss SEM (ZEISS EVO) instrument from Germany was used to examine the morphological properties of the DRH and DRH-MoS_2 composites at an accelerated voltage of 10 kV. The samples were carefully mounted on double carbon tape before being coated with a

layer of gold and palladium using a desk sputter coater (DSR1, United Kingdom). For 200 seconds, the coating process was carried out under a vacuum of 200 torrs. Image J software was used to determine the particle size distribution of the MoS_2 component. Further, Energy Dispersive Spectroscopy (EDXS, USA) was used to analyse the elements present on the sample surfaces (at an accelerated voltage of 10 kV) and mapped using Team Basic software for studying the distribution of in-situ grown MoS_2 within the DRH.

Adsorption kinetics

The kinetic model is a mathematical representation of the relationship between the extent of contact and the amount of adsorbate adsorbed onto the adsorbent. Pseudo-first-order kinetic (eq.5) and pseudo-second-order kinetic (eq.6) models were used to investigate the interaction of the solid adsorbent (DRH and DRH-MoS_2) and the liquid adsorbate (MG dye), as well as calculate the rate of adsorption. The active sites on the adsorbent have the most significant influence on the adsorption rate, providing insights into the behaviour and mechanism of the adsorption process. Both models' linear equations are provided below.

$$l\,og(Q_e - Q_t) = \log Q_e - k_1 t \qquad \text{(eq.4)}$$

$$\frac{t}{Q_t} = \frac{1}{k_2 Q_e^2} + \frac{1}{Q_e} t \qquad \text{(eq.5)}$$

Where Q_e and Q_t (mg/g) is the amount of MG (mg) adsorbed on DRH and DRH-MoS_2 at equilibrium and at any time (t), k_1 (min^{-1}) and k_2 ($g\ mg^{-1}\ min^{-1}$) are the rate Pseudo-first-order kinetic and pseudo-second-order kinetic models respectively.

Adsorption isotherm

Adsorption primarily concerns transferring dyes from a liquid phase (aqueous solution) to a solid phase (adsorbent). The transfer process can be described mathematically using equilibrium adsorption isotherms, which present the interaction between the dye and the

adsorbent surface. As a result, Langmuir and Freundlich isotherms (eq. 6 and 7) were investigated, and mathematical representations of both isotherms are provided:

$$\frac{C_e}{Q_e} = \frac{1}{Q_0 b} + \frac{1}{Q_0} C_0 \quad \text{(eq.6)}$$

$$\log Q_e = \log K_F + \frac{1}{n} \log C_e \quad \text{(eq.7)}$$

where Q_0 (mg/g) and b is the adsorption constant or the maximum adsorptive capacity for Langmuir isotherm and adsorption rate respectively. K_F is Freundlich isotherm constant and n is the sign for describing the favourability of the adsorption process.

Adsorption thermodynamic

Adsorption thermodynamics was used to determine the characteristics of the adsorption process, such as whether it was endothermic or exothermic and whether it followed a spontaneous or non-spontaneous mechanism. The thermodynamic properties of the adsorption process, such as Gibbs free energy ($\Delta G°$), enthalpy ($\Delta H°$), and entropy ($\Delta S°$), were calculated using equation (9) and equation (10), and a plot of lnk_c against $1/T$ was generated. The universal gas constant (R) is 8.314 J/mol. K and the absolute temperature expressed in Kelvins (T) were used in these calculations.

$$\Delta G^{\circ} = -RT \ln k_c \quad \text{(eq.8)}$$

$$lnk_c = \frac{\Delta S^{\circ}}{R} - \frac{\Delta H^{\circ}}{RT} \quad \text{(eq.9)}$$

UV-visible diffused reflection spectra (DRS)

The UV-visible diffused reflection spectra (DRS) analysis of DRH- MoS_2 was conducted utilizing the Lambda 750 spectrophotometer, PerkinElmer, USA. The determination of the band gap for DRH-MoS_2 was carried out using Tauc equation (eq.12).

$$\alpha h\nu = A(h\nu - E_g)^n \quad \text{(eq. 10)}$$

where, 'α' represents the absorption coefficient, '$h\nu$' corresponds to photon energy, 'E_g' signifies the bulk optical band gap, and 'n' is assigned a value of 2 for an indirect band gap. 'A' remains a constant in the equation.

Antioxidant activity of the DRH-MoS_2

The DPPH free radical scavenging experiments were performed according to the literature by Hong et al.[1]. Firstly, 0.3 Mm DPPH was prepared in ethanol. Different concentrations of DRH-MoS_2 (0.1-0.25 mg/ml) were prepared in both ethanol and distilled water and sonicated for 1 min at 27°C. 0.5 mL of prepared DRH-MoS_2 samples were mixed in 0.5 mL DDPH solution and incubated in the dark for 30 minutes. The percentage of DDPH inhibition of the sample was calculated using equation 11.

$$DPPH\ inhibition\ percent = \left[1 - \left(\frac{A_s - A_b}{A_c}\right)\right] \times 100 \quad \text{(eq.11)}$$

The absorbance of DPPH in ethanol is represented as A_c, while A_b signifies the absorbance of the sample in the absence of DPPH, and A_s is the absorbance of the sample in the presence of DPPH after a 30-minute dark incubation.

Table S1: Represents the elemental composition of DRH and DRH-MoS_2 determined through XPS spectroscopic studies.

**DRH-MoS₂**	
**Element**	**Atomic (%)**
**C**	50.05
**O**	38.66
**Si**	6.89
**S**	3.36
**Mo**	1.05
**DRH**	
**C**	53.3

**O**	36.78
**Si**	9.9
**S**	-
**Mo**	0.02

Table S2: Crystal structure properties of RH, DRH and DRH-MoS_2 determined through XRD.

Name of sample	2θ	C.I.	Crystallite size (in nm)	d-spacing (in nm)
**RH**	16.4	45.63%	0.039	0.532
	22.2		0.023	0.404
**DRH**	16.4	51.30%	0.04	0.537
	22.2		0.027	0.401
**DRH-MoS_2**	16.4	30%	0.012	0.514
	22.2		0.0190	0.401

Table S3: Represents pseudo first-order and pseudo second order kinetic parameters for the adsorption of MG onto DRH.

Concentration (mg/L)	Pseudo-first-order kinetic		Pseudo-second-order kinetic	
	K_1	R^2	K_2	R^2
**100**	0.01221	0.99	0.000872	0.88
**200**	0.00599	0.94	0.000378	0.92
**400**	0.01245	0.98	0.003087	0.99
**600**	0.01147	0.98	0.000162	0.77
**1000**	0.03157	0.95	0.00024	0.81

Langmuir isotherm			Freundlich isotherm		
R^2	B	Q_0	R^2	K_F	*1/n or n*
**0.98979**	0.017714	80.45	0.91661	2.25	0.40 or 2.5

Table S4: Details of the adsorption isotherm parameters for DRH

Table S5: Represents the adsorption thermodynamic parameter for DRH

Temperature	ΔG°	ΔS°	ΔH°
**303.13**	- 2.16	65.89 kJ/mol	0.22 kJ/K.mol
308.13	-3.38		
313.13	-6.88		
318.13	-7.19		
323.13	-10.58		

Table S6: List of pseudo first-order and pseudo second-order kinetic parameters of DRH-MoS_2 for photodegradation of MG at different concentrations.

**Concentration (mg/L)**	**Pseudo-first-order kinetic**		**Pseudo-second-order kinetic**	
	K_1	R^2	K_2	R^2
**100**	0.06245	0.81	0.000441	0.99
**200**	0.08172	0.87	0.008768	0.99
**400**	0.03163	0.75	0.016214	0.99
**600**	0.04246	0.82	0.015195	0.99
**1000**	0.07917	0.96	0.067943	0.99
**2700**	0.05461	0.93	0.051987	0.99

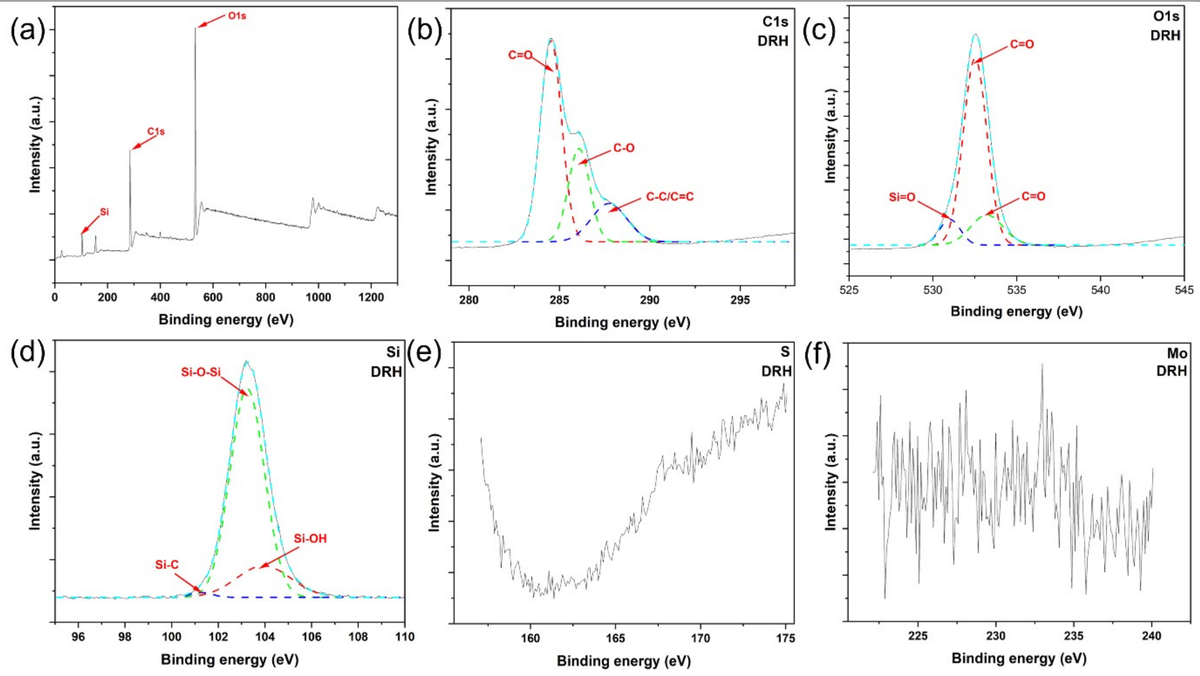

Figure S1: XPS survey a broad spectrum of DRH, with a small scan section of (d) C1s, (e) O1s, (f) Si2p, (g) S2p and (h) Mo3d of DRH.

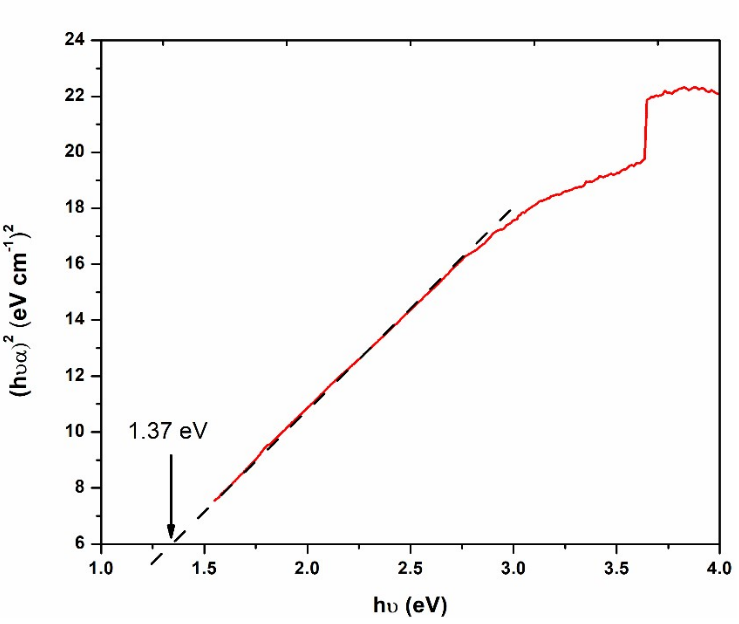

Figure S2: UV-visible DRS spectra of DRH-MoS_2 catalyst.

Effect of time on the formation of MoS_2 on DRH

MoS_2 gown at the optimized temperature reported in our previous study using sugarcane biomass at 195°C for 18 hours was selected for this study with slight modification. On utilizing DRH as substrate for *in-situ* synthesis of MoS_2, we found that after 17 hours of reaction at 195°C, the white colour of DRH turn to grey and in the SEM image sheet or flower like morphologies was not discovered. However, after18 hours of reaction at 195°C, DRH colour turns to light black but still the nanosheets or nanoflower like morphologies were absent in the SEM micrographs. After 19 hours of reaction at 195°C, DRH turns to complete black and nanoflower like morphologies were observed in SEM micrographs of MoS_2 (**Figure S3**). From these results we confirmed the growth and formation of MoS_2 nanoflowers on to the microstructures of DRH which occurs at 195°C for 18 hours.

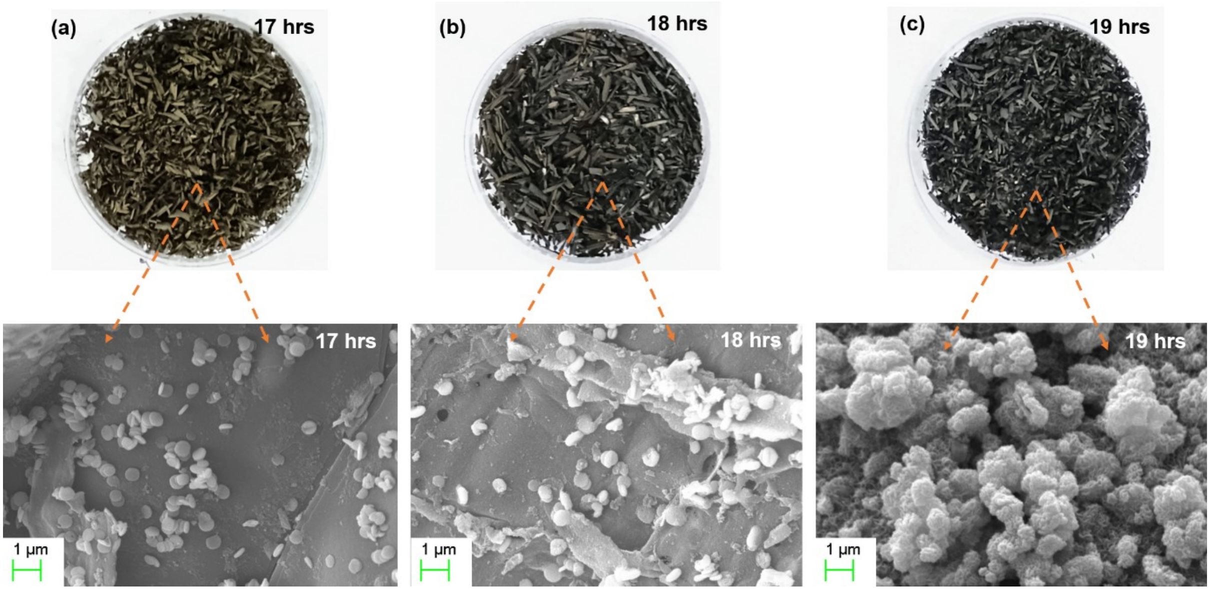

Figure S3: Represent the photograph and SEM image of samples prepared at 195°C (a) 17 Hrs, (b) 18 Hrs, and (c) 19 hrs.

Table S7: FTIR table of RH, DRH, and DRH-MoS_2

Wavenumber (cm^{-1})	Functional group	Ref.
796	Si-O	[2]
1066	Si−O−Si stretching vibrations	[3]
1632	C=O stretching vibration	[4]
3425	Bending vibration of -OH	[5]
2896	C-H stretching	[6]
1616	C=C bond of aromatic carbon of lignin	[7]
1736	C=O stretching of acetyl groups	[8]
560	Mo-S bond	[9]
664	O-Mo-O bond	[10]
1107	S-S bond	[11]
1432	C-H bond	[12]

Table S8: Comparison of present work and summary of dye degradation capabilities of MoS_2 and its composite from literature, encompassing details on dosage quantity, percent degradation of dyes, process parameters and duration.

Catalyst	Reaction time and temperature	Catalyst used	Dye and concentration	Removal efficiency	Time (min)	Ref.
**MoS_2**		5 g	Rhodamine-B (20 mg/L)	96%	40 min	[13]
**$MoS_2/Mg(OH)_2/BiVO_4$**	180 °C for 24 h	0.08 g	Congo red and MG (20 ppm)	95% and 97%	60 min	[14]
**MoS_2**	24 hrs, 180°C	0.4 g/L	Rhodamine-B (20 ppm)	100%	30 min	[15]
**PANI-CS @MoS_2**		0.1 g	Rhodamine-B (30 mg/L) and MG (50 mg/L)	96.2% and 91.5%	60 min	[16]
**BC/MoS_2**	4 hrs, 121°C	5 mg/L	MG 100 mmol	95%	10 min	[17]
**MoS_2**	24 hrs, 180°C	10 mg	Methylene blue (20 mg/L)	98%	30 min	[18]
**gC_3N_4@CS-MoS_2**	24 hrs, 180 °C	60 mg	Rhodamine-B (50 mg/L)	99.6%	50 min	[19]
**3D-$MoS_2/FeCo_2O_4$**	12 hrs, 110 °C	-	Rhodamine-B (30 mg/L)	89.3%	10 min	[20]
**MoS_2–FeS_2**	24 hrs, 200 °C	2.5 mg	Methylene blue (20 mg/L)	100% upto 500 mL	30 min	[21]
**Cel/MoS_2**	16 hrs, 210 °C	10 mg	Rhodamine-B (10 µM)	~ 97%	30 min	[22]
**M-G-CFP**	24 hrs, 200 °C	30 mg	Methylene blue (100-300 mg/L)	94%	400 min	[23]
**BC/MoS_2 hybrid aerogel**	24 hrs, 140-215°C	-	Methylene blue (100-300 mg/L)	96%	120 min	[24]
**3D cellulose-MoS_2 aerogel**	24 hrs, 180 °C	250 mg	Congo red	334.92 mg/g	60 min	[25]
**FSC-wood**	24 hrs, 180 °C	0.6 mg/L	Tetracycline (20 mg/L)	100 %	120 min	[26]
**DRH-MoS_2**	20 hrs, 195 °C	200 MG	MG	100% (upto 500 mg/L)	240 min	This work

Table S9: Represent free radical scavenging activity of DRH-MoS_2

DRH-MoS_2 concentration (mg/L)	DPPH radical scavenging activity (%)
**0.05**	43.52
**0.1**	49.87
**0.15**	57.71
**0.2**	66.75
**0.25**	74.20

References

1 L. Hong, J. Li, F. Liu, S. Huang, B. Zheng, X. Ma, Q. Zhang, B. Zhao and C. Yang, *Colloids and Surfaces A: Physicochemical and Engineering Aspects*, 2020, **596**, 124722.
2 W. Guo, G. Li, Y. Zheng and K. Li, *RSC Advances*, 2021, **11**, 34915–34922.
3 F. Unglaube, C. R. Kreyenschulte and E. Mejía, *ChemCatChem*, 2021, **13**, 2583–2591.
4 S. Azat, A. V. Korobeinyk, K. Moustakas and V. J. Inglezakis, *Journal of Cleaner Production*, 2019, **217**, 352–359.
5 Y. Li, Y. Zhou, R. Wang, Z. Chen, X. Luo, L. Wang, X. Zhao, C. Zhang and P. Yu, *Food Chemistry*, 2022, **389**, 132987.
6 T. N. Ang, G. C. Ngoh, A. S. M. Chua and M. G. Lee, *Biotechnology for Biofuels*, 2012, **5**, 67.
7 S. Yue, C. Cui, K. Wei, Y. Jiang, Z. Bai, J. Ma, C. N. K. Patabendige and C. Jiang, *International Journal of Energy Research*, 2022, **46**, 9822–9835.
8 L. S. Monte, V. A. Escócio, A. M. F. de Sousa, C. R. G. Furtado, M. C. A. M. Leite, L. L. Y. Visconte and E. B. A. V. Pacheco, *Biomass Conv. Bioref.*, 2018, **8**, 189–197.
9 M. Maqsood, S. Afzal, A. Shakoor, N. A. Niaz, A. Majid, N. Hassan and H. Kanwal, *J Mater Sci: Mater Electron*, 2018, **29**, 16080–16087.
10 D. Tang, J. Li, Z. Yang, X. Jiang, L. Huang, X. Guo, Y. Li, J. Zhu and X. Sun, *Chemical Engineering Journal*, 2022, **428**, 130954.
11 S. Singh, S. Sharma, B. S. Bajwa and I. Kaur, *Journal of Environmental Chemical Engineering*, 2022, **10**, 108883.
12 N. T. Nguyen, N. T. Tran, T. P. Phan, A. T. Nguyen, M. X. T. Nguyen, N. N. Nguyen, Y. H. Ko, D. H. Nguyen, T. T. T. Van and D. Hoang, *Journal of Industrial and Engineering Chemistry*, 2022, **108**, 150–158.
13 B. Joshi, A. M. E. Khalil, S. Zhang, F. A. Memon and Z. Yang, *ACS Eng. Au*, 2023, **3**, 461–476.
14 D. Karthigaimuthu, S. Ramasundaram, P. Nisha, B. Arjun Kumar, J. Sriram, G. Ramalingam, P. Vijaibharathy, T. H. Oh and T. Elangovan, *Chemosphere*, 2022, **308**, 136406.
15 R. Mohammed, M. E. M. Ali, S. M. Abdel-Moniem and H. S. Ibrahim, *Nano-Structures & Nano-Objects*, 2022, **31**, 100900.
16 P. Sirajudheen, S. Vigneshwaran, V. C. R. Kasim, M. C. Basheer and S. Meenakshi, *International Journal of Biological Macromolecules*, 2023, **249**, 126008.
17 H. Shen, S. Liao, C. Jiang, J. Zhang, Q. Wei, R. A. Ghiladi and Q. Wang, *Carbohydrate Polymers*, 2022, **277**, 118853.
18 M. T. Leng Lai, K. Mun Lee, T. C. Kuang Yang, G. Ting Pan, C. Wei Lai, C.-Y. Chen, M. Rafie Johan and J. Ching Juan, *Nanoscale Advances*, 2021, **3**, 1106–1120.
19 M. Nikitha, S. SD. Elanchezhiyan and S. Meenakshi, *Environmental Research*, 2023, **238**, 117032.
20 C. Huang, H. Liu, C. Sun, P. Wang, Z. Tian, H. Cheng, S. Huang, X. Yang, M. Wang and Z. Liu, *Environmental Pollution*, 2023, **325**, 121391.
21 H. Ashrafi, F. Rahnama, M. Akhond and G. Absalan, *Inorg. Chem.*, 2022, **61**, 1118–1129.
22 N. Tavker and M. Sharma, *Journal of Environmental Management*, 2020, **255**, 109906.
23 A. Gopalakrishnan, S. Pratap Singh and S. Badhulika, *New Journal of Chemistry*, 2020, **44**, 5489–5500.
24 E. P. Ferreira-Neto, S. Ullah, T. C. A. da Silva, R. R. Domeneguetti, A. P. Perissinotto, F. S. de Vicente, U. P. Rodrigues-Filho and S. J. L. Ribeiro, *ACS Appl. Mater. Interfaces*, 2020, **12**, 41627–41643.

25 S. Qiu, H. Zhang, M. Sang, F. Liu and G. Nie, *Water Air Soil Pollut*, 2023, **234**, 401.
26 W. Zhang, X. Wang, D. Wang, J. Shu, Z. Ye, X. Zhang, L. Ran, Q. Zhao, B. Zou and L. Zhou, *Cellulose*, 2022, **29**, 5079–5101.

88
